# A multicentre study reveals dysbiosis in the microbial co-infection and antimicrobial resistance gene profile in the nasopharynx of COVID-19 patients

**DOI:** 10.1038/s41598-023-30504-3

**Published:** 2023-03-13

**Authors:** A. Sayeed. M. Mahmud, Christine A. Seers, Aftab Ali Shaikh, Tarannum Taznin, Mohammad Samir Uzzaman, Eshrar Osman, Md. Ahashan Habib, Shahina Akter, Tanjina Akhtar Banu, Md. Murshed Hasan Sarkar, Barna Goswami, Iffat Jahan, Chioma M. Okeoma, Md. Salim Khan, Eric C. Reynolds

**Affiliations:** 1 Bangladesh Council of Scientific and Industrial Research, Dr. Qudrat-E-Khuda Road, Dhaka, 1205 Bangladesh; 2 The Oral Health Cooperative Research Centre, Melbourne Dental School, Bio21 Institute, The University of Melbourne, Parkville, VIC 3010 Australia; 3 Jashore University of Science and Technology, Jashore, 7408 Bangladesh; 4 SciTech Consulting and Solutions, Dhaka, 1213 Bangladesh; 5 Department of Pathology, Microbiology, and Immunology, New York Medical College, 40 Sunshine Cottage Rd, Valhalla, NY 10595 USA

**Keywords:** Viral infection, Bacterial infection

## Abstract

The impact of SARS-CoV-2 infection on the nasopharyngeal microbiome has not been well characterised. We sequenced genetic material extracted from nasopharyngeal swabs of SARS-CoV-2-positive individuals who were asymptomatic (*n* = 14), had mild (*n* = 64) or severe symptoms (*n* = 11), as well as from SARS-CoV-2-negative individuals who had never-been infected (n = 5) or had recovered from infection (n = 7). Using robust filters, we identified 1345 taxa with approximately 0.1% or greater read abundance. Overall, the severe cohort microbiome was least diverse. Bacterial pathogens were found in all cohorts, but fungal species identifications were rare. Few taxa were common between cohorts suggesting a limited human nasopharynx core microbiome. Genes encoding resistance mechanisms to 10 antimicrobial classes (> 25% sequence coverages, 315 genes, 63 non-redundant) were identified, with β-lactam resistance genes near ubiquitous. Patients infected with SARS-CoV-2 (asymptomatic and mild) had a greater incidence of antibiotic resistance genes and a greater microbial burden than the SARS-CoV-2-negative individuals. This should be considered when deciding how to treat COVID-19 related bacterial infections.

## Introduction

Severe acute respiratory syndrome coronavirus-2 (SARS-CoV-2) is the virus responsible for coronavirus disease 2019 (COVID-19), a condition with a diverse range of pathological changes, our understanding of which is continually evolving^1–3^. Secondary viral, fungal, and bacterial infections in people with SARS-COV-2 infection can influence disease progression and death rate, thus affecting prognosis^4–9^. The reported SARS-CoV-2 co-infection rates with pathogenic microorganisms vary from less than 1% up to 50%. This variance is likely due to testing regimens, with some patient samples tested during hospitalization with culture confirmation and other samples tested retrospectively using molecular methods^10,11^, with no confirmation of disease- relating to identifications. The rate of co-infection in COVID-19 patients increases with time of nosocomial exposure^12^. Case studies and large-scale surveillance analyses of various respiratory pathogens have shown SARS-CoV-2 co-infection with *Mycobacterium tuberculosis*, dengue, and influenza virus^13–15^ and it has been reported that 50% of COVID-19 deaths were associated with bacterial and fungal co-infections^16^. However, it is often not confirmed if coinfecting species were also causing an associated disease pathology, morbidity and mortality.

Antibiotic therapies are frequently used prophylactically with hospitalized COVID-19 patients however there are contraindications for use of antibiotics as a treatment^12,17^. Survey of patients admitted to hospital with COVID-19 showed bacterial co-infection was uncommon, yet the use of antibiotics was high with lower survival rates in patients given antibiotics, except for those prescribed macrolides^12^. A retrospective analysis of antibiotic use by COVID-19 patients in the two years prior to admission to hospital showed poorer outcomes relative to patients who had no prior use of antibiotics ^17^. Although the advantage of the intake of antibiotics during a viral respiratory infection is that individuals can protect themselves against secondary bacterial infection, conversely antibiotic intake may cause microbiome dysbiosis by repressing health-associated species and allowing the emergence of antibiotic-resistant pathogens^18^. The upper respiratory tract (URT) microbiota is known to be a gatekeeper of respiratory health, preventing or resisting the intrusion of invasive respiratory pathogens^19^, including opportunistic pathobionts ^20^ such as *Streptococcus pneumoniae*^21^, *Haemophilus influenzae* ^22^, *Neisseria meningitidis*^23^ and *Staphylococcus aureus* ^24^ which may exist as harmless commensals or as highly invasive and deadly pathogens. It was suggested that respiratory infections are associated with a nasopharyngeal microbiota imbalance^25,26^ with pneumonia in both old and young adults due to the overgrowth of a single species in the URT and the absence of distinct anaerobic bacteria^26^.

Prior to the emergence of SARS-CoV-2 acute respiratory infections, especially pneumonia, were among the leading causes of death. Worldwide in 2016 alone there were 2,377,697 pneumonia-associated deaths reported^27,28^. *S. pneumoniae* was the leading cause of lower respiratory tract infection, morbidity and mortality causing more deaths than all other aetiologies combined in 2016^27^. Understanding the relationships between organisms such as *S. pneumoniae* and SARS-CoV-2 when they co-inhabit the nasopharynx and lungs would be important to understanding the progression of disease in a COVID-19 patient. On 8 March 2020, the first coronavirus case was reported in Bangladesh and by January 2022 more than 1.5 million individuals were confirmed as infected, with over 28,000 deaths (https://covid19.who.int/region/searo/country/bd). In developing countries such as Bangladesh antibiotics can be obtained without the requirement for a prescription from a medical practitioner which leads to widespread ad hoc intake. Analysis of the prescribing practices in Bangladesh revealed that 83% of patients who visited doctors were prescribed two or more antibiotics without the benefit of laboratory testing indicating a need for such a therapy^25,29^. Unfortunately half of the patients usually stop taking the antibiotic as soon as symptoms abate^30^. This self-medication with antibiotics combined with over-prescription may be contributing to the rising occurrence of anti-microbial resistant bacteria in Bangladesh^31^. In the context of treatment for COVID-19, the prevalence of antimicrobial resistance genes within the microbiome could negatively impact treatment outcomes for patients.

The aim of the present study, was to use DNA sequencing to determine if SARS-CoV-2 infection impacts the nasopharynx microbiome and the profile of antimicrobial resistance genes..

## Materials and methods

### Patients, controls, and sample collection

This study was approved by the National Institute of Laboratory Medicine & Referral Centre (NILMRC) Dhaka, Bangladesh; approval number NILMRC/2020/001. The study methods were carried out in accordance with the relevant guidelines and regulations and complied with the National Statement on Ethical Conduct in Human Research 2007 (Updated 2018). All participants and/or their legal guardians gave informed, written consent for the nasopharyngeal swab samples to be used in the study. Samples were processed at the Genomic Research Lab, Bangladesh Council for Scientific and Industrial Research (BCSIR), Dhaka, Bangladesh. Nasopharyngeal specimens were taken from suspected COVID-19 patients and volunteer control subjects in the cities of Narayanganj, Dhaka, and Chattagram^32^. Specimens were kept at 4 °C before and during transport when sampled and processed on the day of collection or stored at −20 °C and transported on ice for processing within 24 h of collection.

### SARS-CoV-2 detection and shotgun sequencing

SARS-CoV-2 in nasopharyngeal swabs was detected following protocols in the Novel Coronavirus (2019-nCoV) Nucleic Acid Diagnostic Kit (Sansure Biotech). Nasopharyngeal swabs were immersed in Sample Storage Solution for transport and storage. On receipt in the laboratory samples were lysed by addition of Sample Release Reagent. An aliquot of this lysis mix was used for real-time reverse transcription polymerase chain reaction (rRT-PCR) to detect the SARS-CoV-2 N- and ORF 1ab transcripts as per the manufacturer instructions. A second aliquot of lysis mix was centrifuged at 4000 × *g* for 5 min. The supernatant was removed to a clean vial and nucleotides extracted using the Purelink Viral DNA/RNA Extraction Kit (Invitrogen) and used to make sequencing libraries. Sequencing libraries were made using a “shotgun” method with the Illumina TruSeq Stranded Total RNA Library Workflow with an average insert size of 151 bp paired end (Illumina Inc., San Diego, CA). Libraries were sequenced with an Illumina NextSeq 550 instrument according to the manufacturer’s protocol.

### Bioinformatic analysis

Bacterial, fungal, and viral species were identified from raw fastq files using the Chan Zuckerberg ID (CZ ID) portal (formerly IDseq)^33,34^. The CZ ID pipeline performs adapter trimming, data quality control (QC), host DNA subtraction, then alignments using Bowtie 2^35^ to match the raw fastq file’s reads to the National Center for Biotechnology Information (NCBI) national nucleotide collection (NT) and translations to the NCBI non-redundant protein (NR) databases. We created a background dataset using SARS-CoV-2-negative control samples (dataset New BCSIR NSP Background 23 01 2021 in CZ ID). By default, CZ ID ranks taxa based on an aggregate score derived from comparisons to NT/NR z scores and reads per million (rpm). However, this method alone was insufficient to distinguish specific taxa from a list of possible pathogens and non-specific taxa for the given samples. To enable pathogen discrimination, we applied criteria of nucleotide reads per million (NT %id ≥ 95, NT rPM) ≥ 40, NR rPM ≥ 40, nucleotide length (NT L) ≥ 100, NR r (total reads) ≥ 40, and NR value E E−15. After selecting the background model, the NT z-score was set to 1 to compare the relative abundance of the taxon in the samples to the background model. To reduce the possibility of reporting taxa that result from kit, handling and environmental contamination, which could become over-represented, particularly in low biomass samples^36^, we set a cut-off of > 0.1% non-host read abundance for taxa used in comparative analyses. Anti-microbial resistance genes (ARG) were detected using software SRST2^37^ on the CZ ID platform. The number of matching and non-matching bases at each location in each ARG alignment was determined with a binomial test against the reference allele with a hit retention threshold set to > 25% percent gene coverage. For comparison, lower stringency filtering at NT rPM ≥ 10, nucleotide length NT L ≥ 50 was also conducted.

### Statistical analysis

Microsoft Excel (Microsoft 365, version 2212) and the statistical programming language R (version 4.2.2; R) were used to conduct the statistical analyses. The R packages used were imported into RStudio (Studio Team (2020). RStudio: Integrated Development for R. RStudio, PBC, Boston, MA, URL http://www.rstudio.com/) from The Comprehensive R Archive Network repository (https://cran.r-project.org/). Heat maps were generated using the R package pheatmap version 1.0.12, (https://CRAN.R-project.org/package=pheatmap) whereas Venn diagrams were drawn using the VennDiagram package, version 1.7.3 (https://CRAN.R-project.org/package=VennDiagram). Microbiome compositions were assessed by Shannon diversity, H = −∑[(pi) * ln(pi)] where pi is the proportion of each species based on read counts; Simpson’s Index, N(N−1)/sum[n(n−1)] where N is all taxa count and n is count by taxon; and evenness, H/ln (number of species). The Wilcoxon signed-rank test was performed using R package vegan to determine differences in values between the species in each group. Pearson correlation (r) was used to calculate linear dependency between pathogens and ARG or all taxa and ARG. The Kendall rank correlation coefficient and Spearman’s rho statistic were used to approximate a rank-based correspondence between pathogens and ARG, or all taxa and ARG. The Kruskal–Wallis H-test was performed to compare continuous variables between groups. Boxplots with overlaid significance in *p*-value format were generated using Microsoft Excel.

### Ethics statement

This study was approved by the National Institute of Laboratory Medicine & Referral Centre (NILMRC) Human Research Ethics Committee Dhaka, Bangladesh. Ethics approval number NILMRC/2020/001. The study methods were carried out in accordance with the relevant guidelines and regulations and complied with the National Statement on Ethical Conduct in Human Research 2007 (Updated 2018). All study participants or their legal guardians gave informed, written consent.

## Results

### Participant demographics

Nasopharyngeal swab samples were obtained from 89 individuals with confirmed SARS-CoV-2 infection as determined using rRT-PCR, with Ct of 16.33 to 29.6 for the N gene and Ct of 18.64 to 29 for ORF 1ab (Supplementary Table 1). The SARS-CoV-2-positive subjects were categorized into three cohorts based on the severity of COVID-19 symptoms, 14 were Asymptomatic, 64 were Mild (including 2 re-infections) and 11 Severe with admission to an intensive care unit (ICU), where two patients died. COVID-19 symptomatic patients had fever along with other symptoms, including cough, anosmia, breathlessness, headache, and malaise. The SARS-CoV-2 Negative cohort (total *n* = 12) was derived from 5 healthy individuals who had never reported symptoms of SARS-CoV-2 infection and who had never tested positive for SARS-CoV-2 by rRT-PCR (Never-infected cohort) and 7 individuals who had previous SARS-CoV-2 infection but had recovered and were subsequently SARS-CoV-2-negative by rRT-PCR screening (Recovered cohort) (Supplementary Table 1).

The majority of the study participants were male (71%) with ages from 9 to 74 years, while the ages of the female participants ranged from 20 to 65 years. The patient age in the Severe group did not substantially vary from the Asymptomatic and Mild (*p* > 0.05); however, male gender bias was more pronounced in the group with a severe disease where 90% of the patients were male. Only two individuals below 20 years of age in the participant cohort were diagnosed with SARS-CoV-2 infection. (Supplementary Table 1).

### Metatranscriptomic sequencing data output

**Figure 1:**
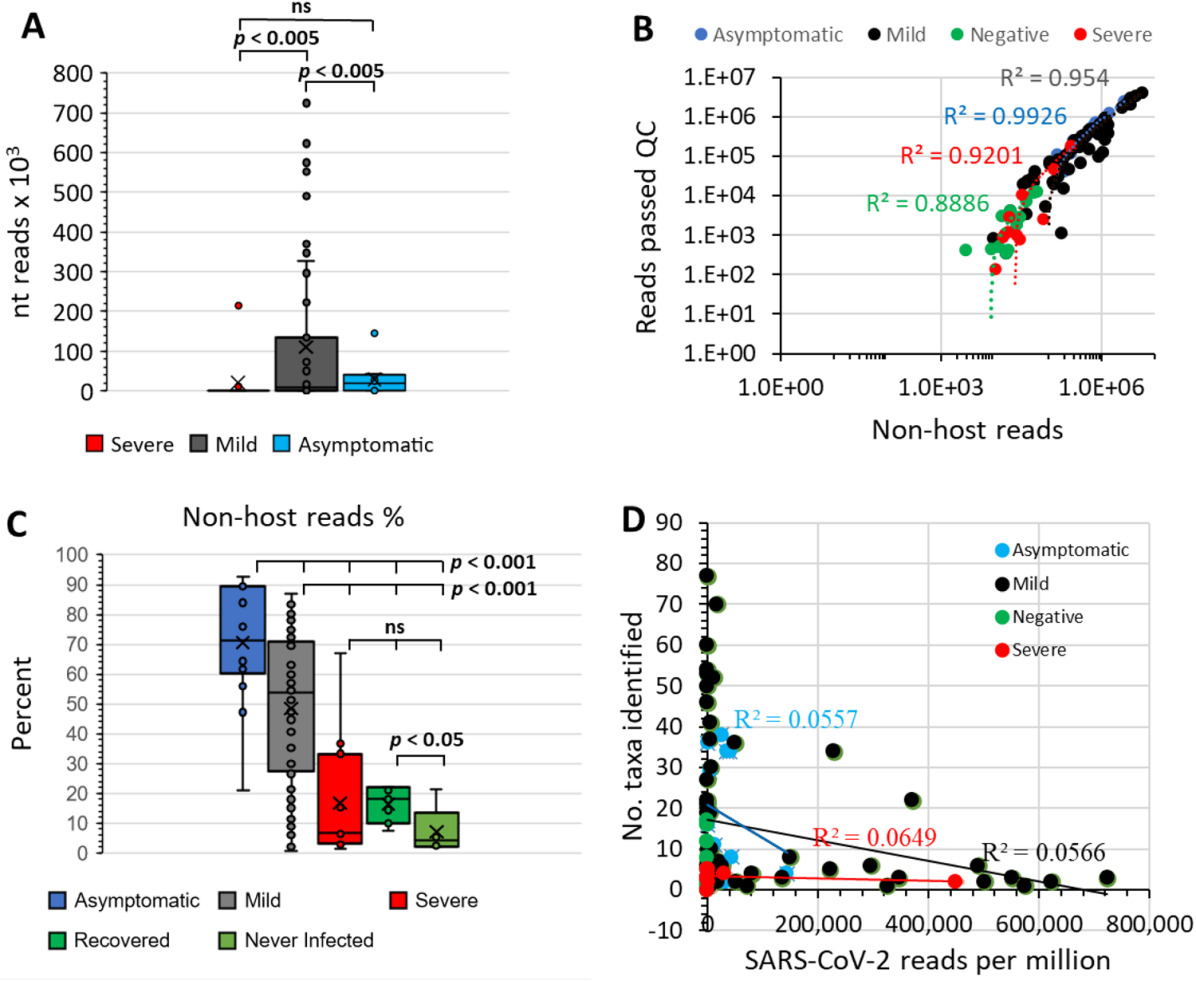
Cohort** s**equencing reads in relation to identification of non-host sequences. (**A**) Comparison of SARS-CoV-2 reads identified relative to COVID-19 symptom severity, showing that the Mild cohort had the highest SARS-CoV-2 loads. Circles indicate the data points. Results of significance comparisons between groups are indicated by *p*-values; ns, not significant. (**B**) Reads that passed final QC, total reads versus non-host reads. High R^2^ values indicate a direct relationship between number of non-host reads and microbiota detection. (**C**) Non-host reads as a proportion of all reads that passed QC for COVID-19 cohorts. SARS-CoV-2 -negative control Recovered and Never Infected cohorts are discriminated. Circles indicate the data points. Results of significance comparisons between groups are indicated by *p*-values; ns, not significant. (**D**) Number of taxa identified in cohorts relative to SARS-CoV-2 reads. Library screening parameters were NT rPM) ≥ 40, nucleotide length (NT L) ≥ 100, and NR value E−15. Low R^2^ values indicate poor linear relationship between SARS-CoV-2 load and identification of taxa. Data was analysed and charts produced in Microsoft Excel version 2212.

The nucleotides extracted from nasopharyngeal swabs were sampled with a reverse transcription step used to convert extracted RNA to cDNA. The DNAs were then used to generate shotgun sequencing libraries that were sequenced using an Illumina NextSeq 550 instrument. Obtained reads per library ranged from 17,724 to 22,501,710 with 83.4% to 99.8% of reads passing QC and no library failing this screening step. Only 76 of 89 samples that were SARS-CoV-2 positive by rRT-PCR had sequences that mapped to SARS-CoV-2, with 9 samples, 2 samples, and 2 samples from the Mild, Asymptomatic and Severe symptom cohorts respectively having no reads discovered. When detected, SARS-CoV-2 reads in the libraries ranged from 2 to 723,812 nucleotides per million total nucleotides sequenced, with an average of 73,918 ± 159,416 (Supplementary Table 1). The number of SARS-CoV-2 sequences identified varied widely within the SARS-CoV-2-positive cohorts but on average was lowest for patients in the Severe group with the Mild cohort having more SARS-CoV-2 reads per sample (*p* < 0.005) than other groups (Fig. 1a). In some libraries more than half of the nucleotides sequenced were assigned to SARS-CoV-2, but for more than 80% of the samples, SARS-CoV-2-derived nucleotides formed less than 5% of the obtained sequences (Supplementary Table 1).

After the final QC the non-host reads (which included SARS-CoV-2 reads) were extracted, revealing as few as 140 and up to 4,047,703 reads per sample in the dataset. Average read abundances per sample were found in the order Mild > Asymptomatic >  > Severe > Negative (Fig. 1b). The Asymptomatic cohort had the highest percentage of all reads per sample being non-host (*p* < 0.001). The Mild cohort also had more non-host reads per sample than the Severe disease and Negative control groups (*p* < 0.001), which were not significantly different from each other. However, the Never Infected group had a lower percentage of non-host sample reads than the Recovered group (Fig. 1c, Supplementary Fig. 1). Comparison of total reads from non-SARS-CoV-2 microbial taxa with SARS-CoV-2 read frequency showed no linear relationships thus, a high SARS-CoV-2 load did not correlate with reduced potential to identify other non-host sequencing reads in the dataset and vice versa (Fig. 1d). Fewest non-host reads per sample were obtained for sequencing libraries produced from the SARS-CoV-2 Negative controls (3,856 ± 4,509) indicating low microbial loads in the sampled material. Patients with Severe COVID-19 symptoms also had low non-host reads per sample (22,673 ± 54,306) indicative of low microbial loads. In terms of total reads, Severe disease samples produced fewer reads than those obtained from Asymptomatic and Mild disease subjects (*p* < 0.005). Notably, the Never-infected negative controls had an absolute lower number of non-hosts reads and a lower percentage of non-host reads relative to total reads than the Recovered group (*p* < 0.05) (Supplementary Table 1) potentially indicating an impact of the prior SARS-CoV-2 infection. Overall, the data suggest the cohort microbiomes accessible to sampling using nasopharyngeal swabs were in the order Asymptomatic > Mild > Severe/Negative.

### The microbiome of the nasopharynx identified within the NGS libraries

We examined the mNGS data for non-SARS-CoV-2 taxa using filter criteria NT rPM ≥ 40, NT L ≥ 100, and NR value E -15. Using this high stringency filter set gave confidence in identifications and limited hits to the more abundant taxa. It has been demonstrated that with low biomass samples, contaminating taxa introduced into the sequencing library from extraction kit materials, the environment and during handling can result in over-representation of contaminants in both 16S and metagenome shotgun sequencing^36^. Therefore, to reduce potential false-positive identifications we set a taxon read abundance cut-off of > 0.1% for comparative analyses. This was particularly important for the Severe and Negative cohorts where total reads obtained for all but one of these 23 samples had raw reads in the lowest 35 samples. There were 17,224–568,692 raw reads, which reduced to 2912–81,153 after QC, indicating low biomass was obtained from these individuals.

**Figure 2:**
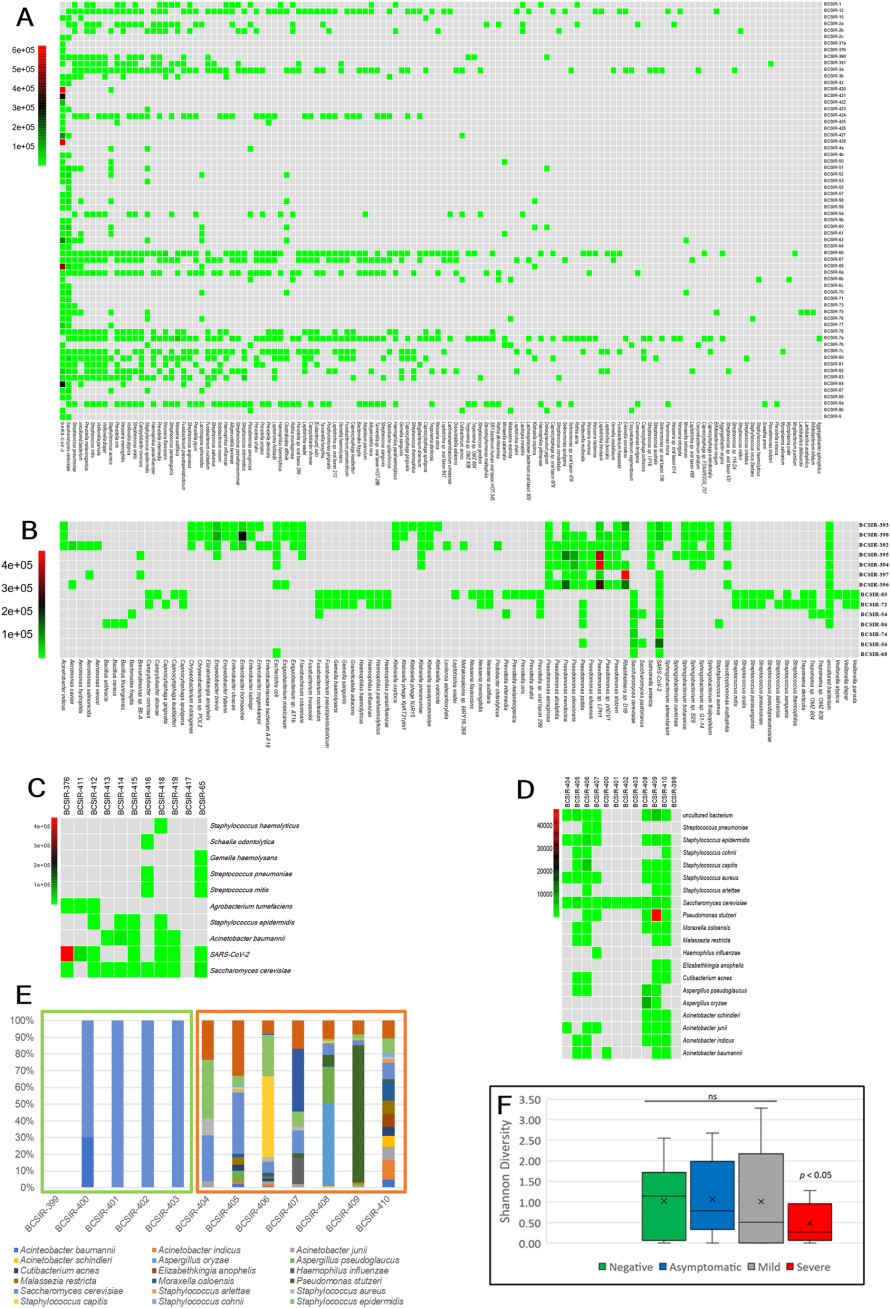
Relative proportions of sequencing reads assigned to species in COVID-19 cohort samples. Heat maps indicate sequences assigned to species identified within (**A**) Mild; (**B**) Asymptomatic; (**C**) Severe; (**D**) Negative cohorts. The heat map was based on the NT rPM values and generated using pheatmap version 1.0.12. The colour coding and range of NT-rPM is indicated to the left of each heatmap (**E**) Stacked plot highlighting taxa identifications in the SARS-CoV-2-negative Never Infected and Recovered cohorts. The light green box indicates Never-infected subjects whilst the orange box indicates Recovered subjects. (**F**) The calculated Shannon diversity index of each cohort. Data was analysed and charts produced in Microsoft Excel version 2212.

In all, there were 1,345 instances of taxa hits, of which 183 were non-redundant (Supplementary Table 2). Excluding SARS-CoV-2 reads from the analysis, reads for each taxon identified in the Negative and Severe cohorts comprised at least 0.1% of reads for that cohort whilst 97.8% and 98.3% of reads for the Mild and Asymptomatic cohorts had abundances ≥ 0.1% (Supplementary Table 2). There were 35 taxa classified for the Severe cohort of which 10 were non-redundant, 956 for the Mild cohort (126 non-redundant), 263 for the Asymptomatic (93 non-redundant), and 91 for the Negative (20 non-redundant) (Table 1). The majority of species identifications in the Mild cohort were obtained from less than one-third of the samples (Fig. 2a), whereas the Asymptomatic cohort species identifications were of reasonably similar numbers for each sample (Fig. 2b). There were some taxa in the Asymptomatic cohort samples with elevated reads relative to other identifications, suggesting colonisation and potential infection. These species were *Rheinheimera* sp. D18, *Pseudomonas* sp. LPH1, *Pseudomonas mendocina*, *Pseudomonas oleovorans* and *Enterobacter hormaechei*. Few taxa were identified from the Severe cohort (Fig. 2c). Notably, more taxa were identified in Recovered individuals than Never-infected, with a potential *Pseudomonas stutzeri* infection in a COVID-19 recovered individual (Fig. 2d, 2e). Shannon diversity calculation supported the indication that overall, the cohorts had even diversity (Fig. 2f, Supplementary Table 3). However, low sequencing reads and subsequent low taxon identifications for the Severe cohort would bias the diversity calculation. In agreement with the suggestion from the heatmaps the Shannon evenness calculation indicated overall moderate evenness in the taxa identifications in the sequencing libraries (Table 2) but this derives from samples between which evenness varies substantially (Supplementary Table 3). Simpson and Shannon diversity values were also calculated, using taxon identifications at lower filter stringencies giving similar findings to that observed with the high stringency filters (Supplementary Table 4). The Simpson diversity index also indicated diversity within cohorts, with the Severe cohort less diverse than the other groups.

**Table 1:** Carriage of taxa, pathogens and ARG in COVID-19 cohorts.

COVID-19 cohort^a^	Total taxa (non-redundant)	Range per sample	Mean	Standard Deviation	Median	Mode	Per sample *p*-value^b^			
Taxa							Severe	ild	Asymptomatic	Negative
Severe	35 (10)	0–5	3.2	1.5	3.2	1.5				
Mild	956 (127)	1–77	14.9	19.4	14.9	19.4	5.3E−06			1.1E-02
Asymptomatic	263 (102)	2–36	18.8	13.2	18.8	13.2	3.3 E−04	1.9 E−01		5.7 E−03
Mild + Asymptomatic	1219 (174)	1–77	15.6	18.4	15.6	18.4	5.1 E−08			3.6 E−03
Negative_All	91 (30)	0–17	7.6	6.7	7.6	6.7	2.3 E−02			
Never Infected	10	0–5	1.0	0.7	1.0	0.7	6.7 E−04	1.60 E−07	1.5 E−04	
Recovered	81	8–17	12.3	4.5	12.3	4.5	6.9 E−04	1.9 E−01	9.3 E−02	
Pathogens										
Severe	15	0–3	1.4	1.1	1.4	1.1				
Mild	605	0–48	9.5	13.2	9.5	13.2	4.4 E−06			7.0 E−02
Asymptomatic	146	0–29	10.4	9.2	10.4	9.2	1.4 E−03	3.7 E−01		7.4 E−02
Mild + Asymptomatic	751	0–48	9.6	12.5	9.6	12.5	9.8 E−08			4.9 E−02
Negative_All	73	0–14	6.1	5.3	6.1	5.3	5.3 E−03			
Never Infected	5	0–2	1.0	0.7	1.0	0.7	2.2 E−01	1.9 E−06	1.3 E−03	
Recovered	68	1–14	9.7	3.7	9.7	3.7	4.1 E−04	4.5 E−01	5.0 E−01	
ARG										
Severe	12	0–2	1.1	0.7	1	1				
Mild	238	0–31	3.7	5.1	1	1	1.0 E−04			3.4 E−07
Asymptomatic	65	0–14	4.6	4.1	4	1	3.5 E−03	2.4 E−01		6.8 E−04
Mild + Asymptomatic	303	0–31	3.9	4.9	2	0	5.1 E−06			2.5 E−09
Negative_All	2	0–1	0.17	0.39	0	0	7.4 E−04			
Never Infected	0	na	na	na	na	na	2.1 E−04	1.0 E−07	6.1 E−04	
Recovered	2	0–1	0.3	0.5	0	0	5.6 E−03	1.1 E−06	1.0 E−03	

Severe, n = 11; Mild, n = 64; Asymptomatic, n = 14; Negative_All, n = 12; Never Infected, n = 5; Recovered, n = 7. Bold text, not significant; Welch’s *t* test, unequal variances; na, not applicable.

**Table 2:** Microbiome diversity in COVID-19 cohorts.

	Negative	Asymptomatic	Mild	Severe
Shannon Diversity (H)	2.02	2.13	3.15	1.22
mean	1.02	1.07	1.01	0.49
Sstdev	0.87	0.88	1.15	0.47
Gamma	20	92	125	9
beta range	0–20	2.49–92	0–125	0–9
Shannon Evenness	0.67	0.47	0.65	0.55
Simpson Reciprocal Index	19.23	76.90	76.08	6.91

	*p*-values Shannon Diversity H			
	Negative	Asymptomatic	Mild	
Asymptomatic	0.444			
Mild	0.484	0.418		
Severe	**0.040**	**0.021**	**0.007**	

Significant values are in [bold].

**Figure 3:**
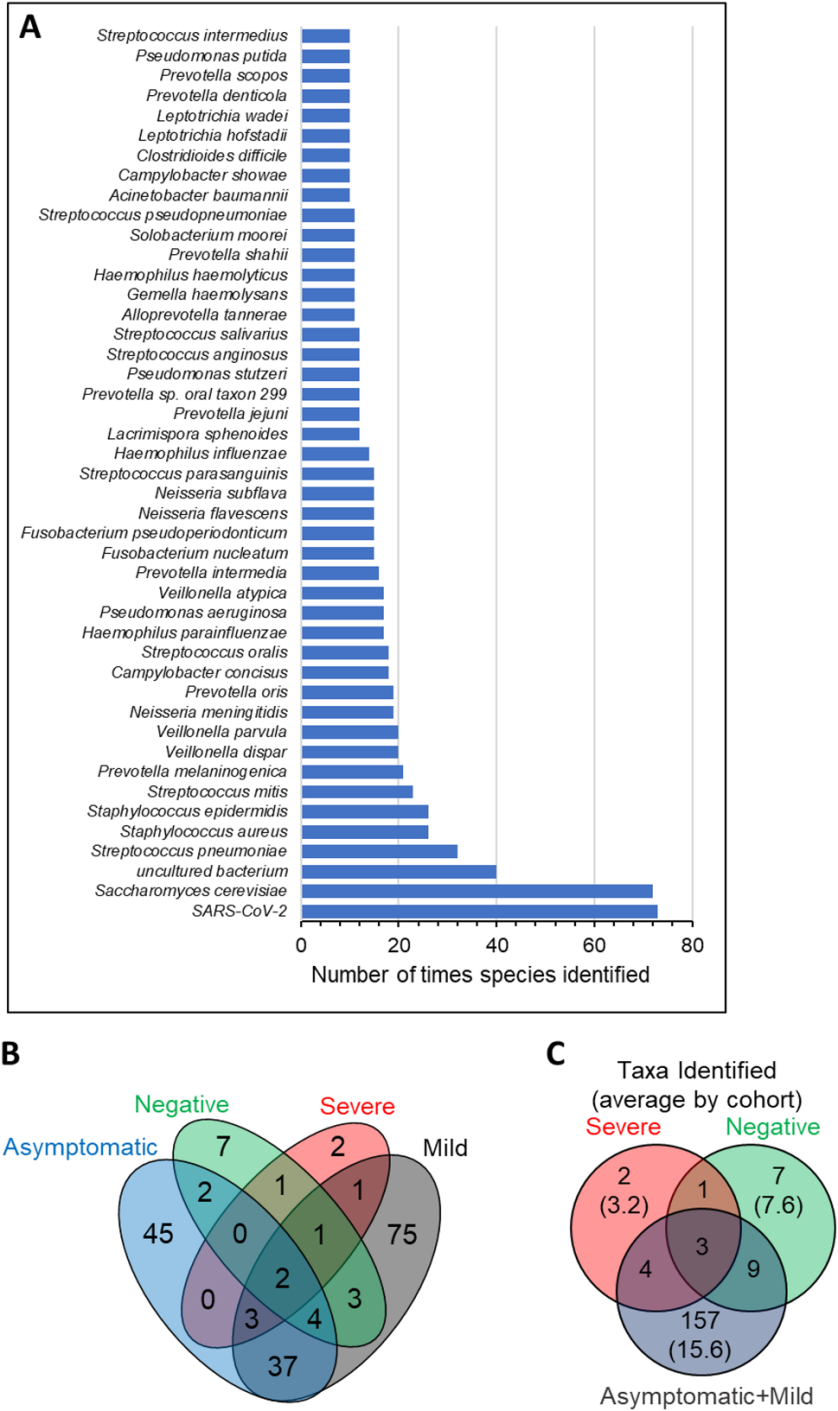
Taxa identified within nasopharyngeal swabs of SARS-CoV-2 cohorts. (**A**) Taxa identified within 10 or more samples. Data was analysed and chart produced in Microsoft Excel version 2212. (**B**) Venn diagram depicting the number of non-redundant taxa of each cohort that are also found within other cohorts. (**C**) Venn diagram depicting of the number of non-redundant taxa common to cohorts with Mild and Asymptomatic cohort data merged. Venn diagrams were generated using VennDiagram version 1.7.3.

Shared taxa between groups were not frequent, for example only 10 taxa were common to 20 or more samples (Fig. 3a) and only *Saccharomyces cerevisiae* and *S. pneumoniae* were identified in all 4 cohorts (Fig. 3b). Excluding SARS-CoV-2, seven taxa occurred in 3 cohorts, an uncultured bacterium, *Staphylococcus epidermidis*, *Streptococcus mitis*, *Haemophilus influenzae*, *Staphylococcus aureus*, *Pseudomonas stutzeri,* and *Gemella haemolysans*. The most frequently identified non-SARS-CoV-2 taxon was *S*. *cerevisiae* with 72 hits. The next most abundant taxon was an uncultured bacterium with 40 classifications, followed by *S. pneumoniae*, *Streptococcus mitis*, *Prevotella melaninogenica*, *Veillonella dispar*, *Veillonella parvula*, *Neisseria meningitidis,* and *Prevotella oris* (Supplementary Table 2). In addition to *S. cerevisiae*, only four fungal species were identified, *Saccharomyces pastorianus, Aspergillus oryzae, Apergillus pseudoglaucus,* and *Malassezia restricta*. Bacteriophages sequences were rare with, only three phage found, *Klebsiella* phage NJR15 (in one sample), *Klebsiella* phage KpKT21phi1 (in two samples) and *Staphylococcus* virus Sextaec in one sample.

Numbers of non-redundant taxa identified in the Asymptomatic cohort (93, range 2–38 per sample) and Mild cohort (127, range 1–77 per sample) were not determined to be significantly different despite different *n* of 14 and 64 respectively, likely due to the large spread in sample taxa identifications between samples (Table 1). Both the Mild and Asymptomatic cohorts had more taxa per sample on average than the Severe and Negative control groups (Table 1; Fig. 3c). When the Negative control group was examined as the subsets of Never Infected and Recovered it was observed that the frequency of taxa identifications in the Recovered cohort was not different to that of the Mild and Asymptomatic cohorts.

**Figure 4:**
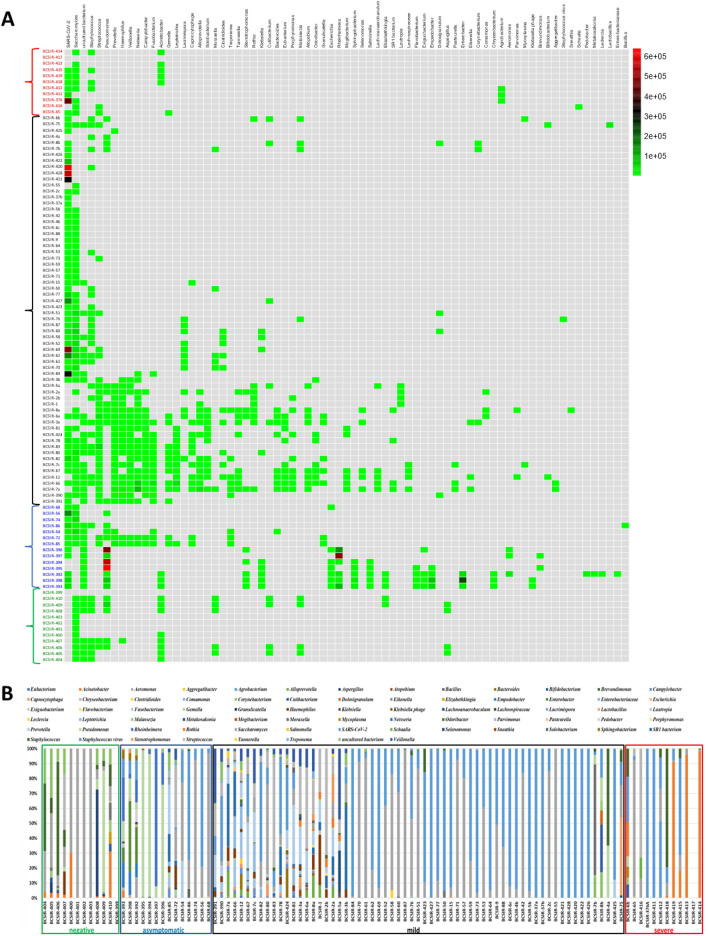
Taxa identified within nasopharyngeal swabs by genera. (**A**) Heatmap indicating sequencing read depths. The heat map was based on the NT rPM values and generated using pheatmap version 1.0.12. The colour coding and range of NT-rPM is indicated to the right side of heatmap. (**B**) Stacked plot of genera. Data was analysed and charts produced in Microsoft Excel version 2212. Cohorts are indicated by the colored sample names (panel A) or boxes (panel B): green, Negative for SARS-CoV-2; blue, Asymptomatic; black, Mild disease; red, Severe disease.

The majority of the non-redundant (excluding SARS-CoV-2) taxa identified (130) were found as singletons occurring in only one sample or one cohort (Supplementary Table 2; Fig. 2 and Fig. 3), an observation which also held using lower stringency filter parameters of NT rPM-10 NT length 50 bp (Supplementary Table 4). Thus, the data suggest microbial diversity both within and between cohorts. At the genus level, there were 73 taxon classifications (Fig. 4a). A stacked plot of the proportions highlights the differences between the cohorts (Fig. 4b).

### Pathogen carriage

Some evidence for pathogenicity was found in the literature for 839 taxa hits of which 98 were non-redundant (Supplementary Table 1). The cohort with severe disease symptoms had a per sample average of significantly fewer taxa identified than the other three groups (*p* < 0.05), which were not significantly different from each other (Table 1). The significantly lower identification of taxa in the Severe cohort may be due to bias induced by prophylactic and therapeutic ICU treatment regimens. This confounding factor could have artificially altered the composition of the microbiota of these subjects. This could be a reflection on the total taxa carried and may also be influenced by antimicrobial treatment in ICU reducing carriage of sensitive taxa.

### Antibiotic resistance genes

**Figure 5:**
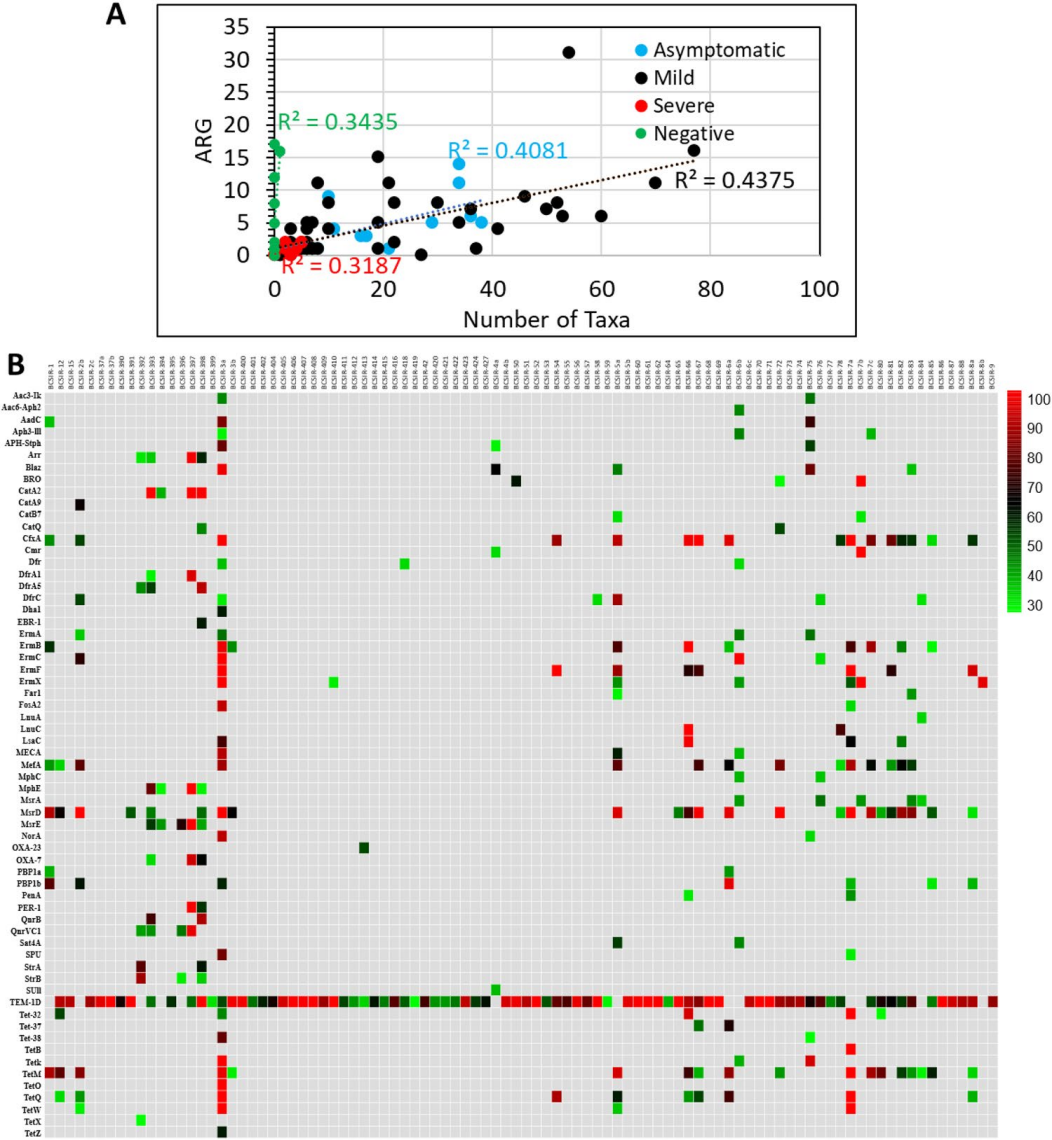
Identification of ARG in COVID-19 cohorts. (**A**) Scatter plot of the number of ARG identified relative to the number of taxa identified by sample. R^2^ indicate poor linear correlations. Data was analysed and chart produced in Microsoft Excel version 2212. Green, Negative for SARS-CoV-2; blue, Asymptomatic; black, Mild disease; red, Severe disease. (**B**) Heatmap showing ARG distribution with sequence coverage > 25%. The heat map was based on the NT rPM values and generated using pheatmap version 1.0.12. The colour coding indicating the percentage sequence coverage is shown to the right of the heatmap. The sample identification number is shown at the top and the identified gene product to the left.

Antibiotic resistance genes (ARG) were identified with > 25% gene coverage in 83 of the 89 SARS-CoV-2 positive samples (Supplementary Table 1). Collectively these genes would impart resistances to 10 antimicrobial classes, including aminoglycosides (Agly), β-lactams (Bla), fluoroquinolones (Flq), fosfomycin (FM), phenicols (Phe), tetracycline (Tet), rifamycin (RM), trimethoprim (Tmt), sulphonamide (Sul), and macrolide-lincosamide-streptogramin (MLS) (Table 3). In all 326 ARG were identified of which 63 were non-redundant genes. There was no relationship between the number of taxa identified within a sample and the number of ARG identified in that sample (Fig. 5a). The predominant non-redundant ARG would impart phenotypes of resistance to β-lactams (16 different genes in 79 samples) followed by MLS (15 genes in 34 samples) and tetracycline (10 genes in 21 samples) (Table 4). More than 10 different ARG with > 25% sequence coverage were identified within many samples, and notably *bla*_TEM-1D_ was in more than 80% of the samples (Fig. 5b). When the more stringent cut-off of excluding genes with ≤ 50% gene coverage was applied, 48 non-redundant genes encompassing 9 classes of ARG were still detected in 80 of the samples (Table 3). Four classes of ARG, including Bla, Phe, Tet, and MLS resistance coding genes were detected in 27 samples with 100% gene coverage (Table 3).

**Table 3:** ARG identified in nasopharyngeal mNGS data.

ARG Product	ARG Class	No. of ARG in samples	Gene Coverage Range (> 25%)	ARG	ARG Class	No. of ARG in samples	Gene Coverage Range (> 25%)
Aac3-Ik	Agly	2	42–45	MefA	MLS	14	30–88
Aac6-Aph2	Agly	1	42	MphC	MLS	2	34–36
AadC	Agly	3	34–81	MphE	MLS	4	26–100
Aph3-III	Agly	3	26–43	MsrA	MLS	5	32–45
APH-Stph	Agly	3	27–78	MsrD	MLS	23	29–100
Arr	RM	4	28–98	MsrE	MLS	5	38–100
BlaZ	Bla	5	34–98	NorA	Flq	2	30–90
BRO	Bla	2	59–100	OXA-23	BL	1	51
CatA2	Phe	4	99–100	OXA-7	BL	3	30–93
CatA9	Phe	1	64	PBP1a	BL	2	36
CatB7	Phe	2	28–30	PBP1b	BL	7	28–96
CatQ	Phe	2	42–52	PenA	BL	2	28–41
CfxA	Bla	16	30–100	PER-1	BL	2	54–99
Cmr	Phe	2	30–99	QnrB	Flq	2	73–87
Dfr	Tmt	3	30–34	QnrVC1	Flq	4	39–97
DfrA1	Tmt	2	27–94	Sat4A	Agly	2	42–53
DfrA5	Tmt	3	41–89	SPU	BL	2	27–80
DfrC	Tmt	6	27–86	StrA	Agly	2	59–76
Dha1	Bla	1	58	StrB	Agly	3	27–86
EBR-1	Bla	1	58	SulI	Sul	1	36
ErmA	MLS	4	33–45	TEM-1D	BL	79	26–100
ErmB	MLS	10	25–100	Tet-32	Tet	5	26–100
ErmC	MLS	4	29–100	Tet-37	Tet	2	45–67
ErmF	MLS	8	68–100	Tet-38	Tet	1	77
ErmX	MLS	7	27–100	TetB	Tet	1	100
Far1	Bla	2	30	TetK	Tet	3	36–100
FosA2	FM	1	90	TetM	Tet	18	26–100
FosB	FM	1	90	TetO	Tet	1	100
LnuA	MLS	1	31	TetQ	Tet	10	30–100
LnuC	MLS	2	73–100	TetW	Tet	4	27–100
LsaC	MLS	4	44–98	TetZ	Tet	1	58
MECA	Bla	3	57–89				

Antimicrobial resistances encoded by ARG: Agly, aminoglycoside; Bla, β-lactam; Flq, fluoroquinolone; FM, fosfomycin; Phe, phenicol; Tet, tetracycline, RM, rifamycin; Tmt, trimethoprim; Sul, sulphonamide; MLS, macrolide-lincosamide-streptogramin.

**Table 4:** Occurrence of non-redundant ARG sequences in COVID-19 cohorts.

ARG Class	Non-redundant ARG with > 25% sequence coverage	Samples with ARG	Non-redundant ARG with > 50% sequence coverage	Samples with ARG
Agly	8	10	5	5
Bla	16	80	12	74
Phe	5	9	4	6
Tmt	4	12	3	5
MLS	15	35	10	24
FM	2	2	1	1
Flq	3	7	3	4
RM	1	4	1	2
Tet	10	21	9	15
Sul	1	1	0	0

n = 101; Antimicrobial resistances encoded by ARG: Agly, aminoglycoside; Bla, β-lactam; Flq, fluoroquinolone; FM, fosfomycin; Phe, phenicol; Tet, tetracycline, RM, rifamycin; Tmt, trimethoprim; Sul, sulphonamide; MLS, macrolide-lincosamide-streptogramin.

## Discussion

The use of metagenome and metatranscriptome sequencing has proven to be an invaluable tool for our understanding of the diversity of microbial communities in relation to health and disease^38–41^, with many uncultured and previously unknown species being identified through these methods^42^. Defining the role of co-infecting bacteria in the pathogenesis of COVID-19 is extremely difficult, despite the prognostic knowledge already obtained for bacterial co-infections that occur during other viral respiratory infections^3,43^. Bacterial and fungal co-infection rates with SARS-CoV-2 are reported to be proportional to disease severity^44^ with increase in death risk ^9,45^ in accord with other viral pneumonias. The microbiome of the nose and nasopharynx in health and disease is poorly understood. In this study, we surveyed the nasopharyngeal microbiomes of healthy individuals, individuals who had active SARS-CoV-2 infection and individuals who had recovered from SARS-CoV-2 infection. Although some samples show species richness many samples show identifications dominated by only a few species and genera regardless of whether an individual is infected with SARS-CoV-2 or not (Fig. 2). The Severe cohort contrasted with the Asymptomatic and Mild cohorts by having fewer taxa identifications. Bias induced by prophylactic and therapeutic ICU treatment regimens could have impacted the composition of the microbiota of these subjects. For this reason, in this study we have put greater emphasis on comparisons between the SARS-COV-2-negative and Mild and Asymptomatic group samples.

There are widely variant alpha and beta diversities with the resultant absence of statistically determined differences between cohorts and evenness in diversity calculations (Table 1, Supplementary Table 2, Supplementary Table 3, Fig. 2). This high between-sample diversity is similar to that found in a recent large-scale 16S metagenome analysis of paired nose and nasopharynx microbiomes of healthy individuals^46^. Both niches were shown to have uneven species distribution of low overall species richness with only a limited number of bacterial genera dominant^46^. Furthermore, distinct flora differences were found between the nasal and pharyngeal niches, with some genera such as *Fusobacterium* and *Streptococcus* never being found in the nose^46^. In the samples examined here, *Streptococcus* was also a dominant genus (32 samples, all 4 main cohorts), as was *Staphylococcus* (37 samples, all 4 main cohorts) and *Pseudomonas* (17 samples, not found in the severe cohort) (Fig. 3, Supplementary Table 2). Interestingly, the Recovered subjects had more diverse microbiomes identified than the Never Infected (Fig. 2) which could be due to an upshift in diversity during infection which has not yet returned to a health-associated low microbiome content or may be a SARS-CoV-2 infection-induced persistent change to the microbiota. Temporal analysis with larger cohort sizes where samples have been obtained from Never Infected, then following subjects during and post infection would prove informative. We found that *Acinetobacter* spp. were frequent in the Severe cohort, as well as recovered individuals but were not identified within the samples from individuals with mild disease, despite the larger sample size (*n* = 64) for this cohort. This could reflect the acquisition of *Acinetobacter* spp, particularly *A. baumannii* as nosocomial infections, which is a growing problem^47^. Hospitalised patients with severe illness are more likely to undergo intrusive interventions, resulting in increased sensitivity to secondary infections, which can make them more vulnerable to nosocomial infection with multidrug-resistant pathogens such as *A. baumannii*, *E. coli*, *P.aeruginosa*, and *Enterococcus spp*.^48^. Infections with similar types of bacterial pathogens have previously been identified in patients with severe influenza^49^.

To understand microbial population shifts as a result of disease via the metagenomic approach it may be more informative to consider only those species of greatest abundance in a sample, suggestive of actual colonization, rather than all species identifiable. The upper respiratory tract (URT) is constantly bathed in airflow from the external environment, which contains 10^4^–10^6^ bacteria per cubic meter of air inhaled^50^. As a result, taxa collected during nasopharynx sampling could be transient microbes or environmental contamination and not established colonizing species^51^. This should be considered when interpreting the importance of taxon identifications within samples. Hoque et al., (2021) recently reported an RNA-seq analysis of nasopharyngeal swab samples from Bangladesh COVID-19 patients (*n* = 8), recovered individuals (*n* = 7), and healthy controls (*n* = 7) reporting identification of a total of 2281 non-redundant bacterial species^52^. They concluded that there was dysbiosis causing a reduced species load in COVID-19 patients. Interrogation of the data of Hoque et al*.,* reveals that the majority of taxa identified each had < 0.1% of the total reads obtained. Combined these low abundance sequences comprised 96.3%, 82.2%, and 92.9% reads in the healthy, COVID-19 and recovered metagenome reads respectively. Thus, the majority of the microbiome species reported in the study were of low abundances (Supplementary Table 5). When we lowered filter stringency, we also identified many taxa which occurred at low abundances (Supplementary Table 3). Filtering the Hoque et al.metatranscriptome data with taxon identifications at ≥ 0.1% of total non-host reads indicates the cohort who had recovered from COVID-19 had the most richness with 91 bacterial species identified (mean 13 per sample), the COVID-19 subjects had 61 species (mean 7.6 species per sample) and the healthy cohort the least richness with 30 species (mean 4.3 per sample). Only *Pseudomonas putida* was shared between all 3 cohorts (maximum abundance 0.47%) (Supplementary Table 5). Combined, the recovered and healthy cohorts (n = 14, the equivalent of our Negative cohort) at ≥ 0.1% abundance had 97 non-redundant species (mean 6.9 taxa per sample) with 24 in common. This agrees with our Negative cohort observation of 91 species of which 20 were in common with a mean of 7.6 ± 6.7 per sample. *Acinetobacter indicus*, *Acinetobacter junii,* and *Pseudomonas stutzeri* were identified in the cohorts in each study. We identified numerous *Acinetobacter* and *Pseudomonas* species within our samples. suggesting that they may be common genera in the oronasal environment. We have previously identified multidrug-resistant *Acinetobacter* sp. and *Pseudomonas* sp. in supragingival plaque in subjects from Pakistan, a country that like Bangladesh has limited restrictions on the use of antimicrobials^53^.

Using the more conservative filter criteria both our, and the Hoque study, suggest on average more taxa in SARS-CoV-2 positive samples than in SARS-CoV-2-negative samples, thus suggesting a dysbiosis in which more species are able to colonize the nasopharynx of SARS-CoV-2 infected individuals. A dysregulated immune system, as is known to be associated with COVID-19^54^ is a likely factor in this dysbiosis. Overall, metagenome/metatranscriptome sequencing of nasopharyngeal swab samples indicated a remarkable diversity in microorganisms that colonise or pass through this niche, however most may have little relevance in the context of COVID-19 and patient outcomes.

The conclusion of Hoque et al., (2021) using their full data set was of dysbiosis causing a reduced species load in COVID-19 patients, which is consistent with the conclusions of Mostafa et al. (2020)^55^. Using Nanopore sequencing Mostafa et al., found indication of coinfection using the criterion of the proportion in the microbiota > 50% above that expected from a healthy individual. The four clinically relevant species were found in 5 of the 40 COVID-19 patient samples examined, 2 viruses, and 2 bacteria *Haemophilus influenzae* (*n* = 2) and *Moraxella catarrhalis*. *Moraxella catarrhalis* was also found in one COVID-19-negative sample. This would indicate potential for coinfection pathology for these individuals albeit at a rate of ~ 12%. However unfortunately, standard-of-care testing was not performed so impacts on patient health from these species are unknown. In view of the beta diversity that we have demonstrated between samples of the same cohort, and noted by others^46^, extrapolation to the conclusion of dysbiosis by examination of small sample sets should be considered with caution. Furthermore, the presence of many singleton identifications can make interpretation of datasets with population sampling of the size used here inaccurate^56^.

Multiple potential confounding factors also need to be considered with the interpretation of the results reported herein. Data collection disparities between the collection sites used prevented confident allocation of matched controls to patients with respect to factors such as antibiotic use and comorbidities. Many subjects, who had volunteered use of their material including those in the Mild cohort, did not require hospital admission so no more than basic demographic data was available. Other confounding factors were that nasopharynx samples were collected by a range of operators with potentially different skill levels and techniques, the behaviour of subjects during collection is not known, other subject health factors such as chronic rhinitis or other URT conditions are not known, urban versus rural living and pollution could impact species exposure and colonisation and so on. Nonetheless, the sequence data provide a useful snapshot of the nasopharyngeal microbiome in a Bangladesh population.

*S. pneumoniae* was one of the most frequently found taxa in our study with all cases in individuals with current SARS-C0V-2 infection or who had recovered from SARS-CoV-2 infection. *S. pneumoniae* is known to increase in both density and frequency in the URT during viral infections with a positive association found between the occurrence of *S. pneumoniae* colonization and the amount of URT pathogens in a pathogen-dependent manner^57^. The prevalence of multidrug-resistant *S. pneumoniae* infection is associated with increased mortality of patients infected with influenza virus^58,59^. The potential for multidrug resistant *S. pneumoniae* infecting patients in our cohorts is of concern for patient outcomes.

*Klebsiella sp*. and *Acinetobacter sp*. were the most common coinfecting bacteria identified in a survey of COVID-19 cohorts in Wuhan, China, , while *Aspergillus* and *Candida* were the most common fungal genera^60^. In our study four species of *Klebsiella* and four species of *Acinetobacter* were identified. Notably, *Klebsiella sp*. were almost exclusively in the Asymptomatic cohort. At least one of the *Acinetobacter sp*. including *A. baumannii, A. indicus*, *A. junii* and *A. schindleri* were in each of the Recovered SARS_CoV-2-negative cohort samples. *A. baumannii* was also found in 5 samples of the Severe cohort and *A. indicus* in 5 samples from the Asymptomatic subjects. However , numerous species were more common, including many species associated with periodontal disease^61^, *Prevotella intermedia* (n = 16), *T. forsythia* (*n* = 8), *T. denticola* (*n* = 7), *Fusobacterium nucleatum* (*n* = 15), and *Porphyromonas gingivalis* (*n* = 8). This may simply indicate saliva contamination rather than nasopharyngeal colonisation because many organisms known to be abundant in saliva, e.g., *Streptococcus salivarius* (*n* = 12), *Streptococcus mitis* (*n* = 23), *Gemella hemolysans* (*n* = 11) and *Rothia mucilaginosa* (*n* = 9)^62^ were also frequently identified. However, it is believed that a range of genera including *Prevotella*, *Sphingomonas*, *Pseudomonas*, *Acinetobacter*, *Fusobacterium*, *Megasphaera*, *Veillonella*, *Staphylococcus*, and *Streptococcus* form part of the healthy lung microbiome, thus the periodontal disease -associated species may indeed be natural colonisers of the nasopharynx in patients with periodontal disease^63–65^.

Our research also explored the potential antibiotic tolerance of the nasopharyngeal swab microbiome in COVID-19 patients. A total of 63 ARGs were detected across 94.4% of the COVID-19 nasopharyngeal samples. The most abundant resistance genes were β-lactams with 15 subtypes, MLS with 14 subtypes, tetracyclines 10 subtypes and aminoglycosides with 10 subtypes. The *bla* (*TEM-1D*) gene is the most prevalent and confers amoxicillin resistance^66^. In Bangladesh, amoxicillin is one of the most prescribed and highest self-medicated antibiotic (10.4%)^67,68^. It has been shown in several studies, (reported in a review^69^) that amoxicillin resistance in *E. coli, Klebsiella* species, and methicillin-resistant *S. aureus* is found with a frequency of 95%, 91%, and 94%, respectively. The random use of antibiotics and resistance shown by several pathogens could explain the high number of *bla (TEM-1D)* genes detected in the Bangladesh nasopharyngeal samples. *S. pneumoniae* can exhibit resistance to multiple antibiotics, but the prevalence rate can differ by region^70^. The most common ARG expressed by *S. pneumoniae* are 6 penicillin-binding proteins (1a, 1b, 2x, 2a, 2b, and 3) that allow β-lactam resistance, and macrolide resistance genes *ermB* and *mefA*^70^. The *ermB* and *mefA* genes were detected in 10 and 14 COVID-19 patients respectively suggesting these genes may have been expressed by this pathobiont in the Bangladesh cohort.

We detected 166 of the 174 bacterial pathogens in only 30 of the COVID-19 patients who also had 64.8% of the detected ARG, thus proportional pathogen and resistance gene prevalence was observed in this subset of patients. The average number of ARGs per overall sample was 3.5 but was 7.2 in those 30 COVID-19 patients with high pathogen carriage. Based on these results, it is interesting to suggestthat the abundance of commensal flora was positively associated with pathobiont and ARG existence, which in turn was positively influenced by SARS-CoV-2 infection.

## Conclusion

Sequencing of the material isolated from the nasopharynx has revealed that individuals infected with SARS-CoV-2 (Mild and Asymptomatic) had more bacterial species in the nasopharynx and more ARG carriage than persons never infected with the virus. This would suggest people infected with SARS-CoV-2 are at greater risk of acquiring a secondary infection, which would be difficult to treat if the infecting organisms carried a multidrug resistance profile.

## Supplementary Information

Supplementary Information 1.

Supplementary Table 1.

Supplementary Table 2.

Supplementary Table 3.

Supplementary Table 4.

Supplementary Table 5.

Supplementary Figure 1.

## Data Availability

The sequencing data generated during this study are available in the CZ ID portal https://czid.org. After requesting access to CZ ID and sign in users can view the data in the public project Co-infection_19_01_2021 which can- also be accessed via the link https://czid.org/public?currentDisplay=table&currentTab=samples&mapSidebarTab=summary&projectId=1402&showFilters=true&updatedAt=2023-01-31T13%3A51%3A49.863Z&workflow=short-read-mngs.
