# Supplementary material for: A multicentre study reveals dysbiosis in the microbial co-infection and antimicrobial resistance gene profile in the nasopharynx of COVID-19 patients
Source: Sci Rep. 2023 Mar 13;13:4122. doi: 10.1038/s41598-023-30504-3 (PMC10009844; doi:10.1038/s41598-023-30504-3)
Supplement: Supplementary file 1 — Supplementary Information 1. [file 41598_2023_30504_MOESM1_ESM.docx]

**INVENTORY OF SUPPLEMENTARY INFORMATION**

**A multicentre study reveals dysbiosis in the microbial co-infection and antimicrobial resistance gene profile in the nasopharynx of COVID-19 patients**

**Supplementary Figure 1**. mNGS read processing of COVID-19 cohort libraries and identification of SARS-CoV-2. (A) CZID pipeline processing of mNGS reads. (B) Total reads and nonhost reads post CZID quality control.

**Supplementary Table 1**. Patient Demographics and NGS read data.

**Supplementary Table 2**. Detected taxa with NT-40 rPM NTL 100bp.

**Supplementary Table 3**. Detected taxa with NT_10 rPM_NTL 50bp.

**Supplementary Table 4**. Diversity calculation.

**Supplementary Table 5**. Hoque 2021 data subset analysis.
