# Supplementary figures and images for: A multicentre study reveals dysbiosis in the microbial co-infection and antimicrobial resistance gene profile in the nasopharynx of COVID-19 patients
Source: Sci Rep. 2023 Mar 13;13:4122. doi: 10.1038/s41598-023-30504-3 (PMC10009844; doi:10.1038/s41598-023-30504-3)

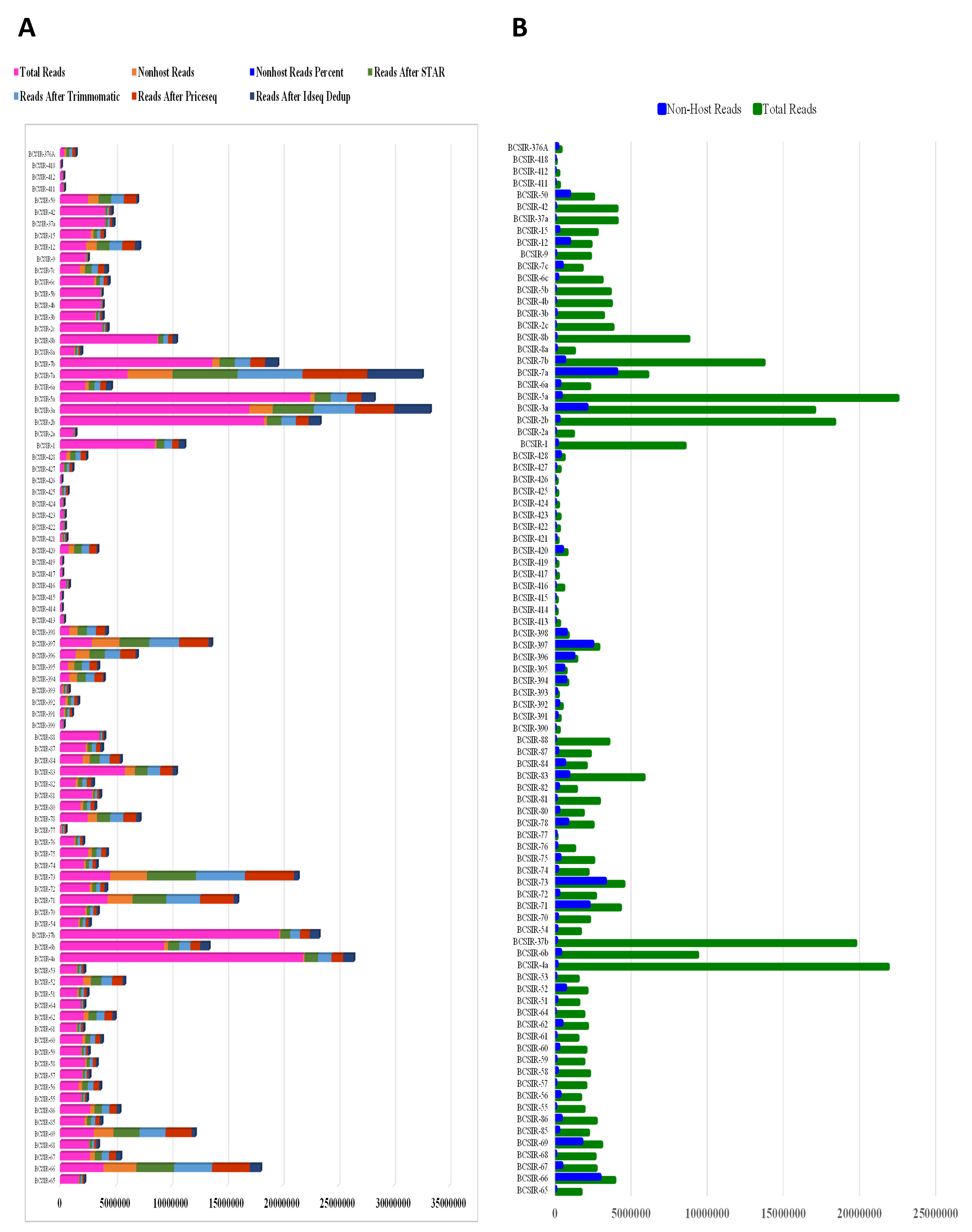

Supplement: Supplementary file 7 — Supplementary Figure 1. [file 41598_2023_30504_MOESM7_ESM.tif]
